# Four simple recommendations to encourage best practices in research software

**DOI:** 10.12688/f1000research.11407.1

**Published:** 2017-06-13

**Authors:** Rafael C. Jiménez, Mateusz Kuzak, Monther Alhamdoosh, Michelle Barker, Bérénice Batut, Mikael Borg, Salvador Capella-Gutierrez, Neil Chue Hong, Martin Cook, Manuel Corpas, Madison Flannery, Leyla Garcia, Josep Ll. Gelpí, Simon Gladman, Carole Goble, Montserrat González Ferreiro, Alejandra Gonzalez-Beltran, Philippa C. Griffin, Björn Grüning, Jonas Hagberg, Petr Holub, Rob Hooft, Jon Ison, Daniel S. Katz, Brane Leskošek, Federico López Gómez, Luis J. Oliveira, David Mellor, Rowland Mosbergen, Nicola Mulder, Yasset Perez-Riverol, Robert Pergl, Horst Pichler, Bernard Pope, Ferran Sanz, Maria V. Schneider, Victoria Stodden, Radosław Suchecki, Radka Svobodová Vařeková, Harry-Anton Talvik, Ilian Todorov, Andrew Treloar, Sonika Tyagi, Maarten van Gompel, Daniel Vaughan, Allegra Via, Xiaochuan Wang, Nathan S. Watson-Haigh, Steve Crouch

**Affiliations:** 1 ELIXIR Hub, Wellcome Genome Campus, Hinxton, CB10 1SD, UK; 2 Netherlands eScience Center, Science Park 140, Amsterdam, 1098 XG, Netherlands; 3 CSL Limited, Bio21 Institute, 30 Flemington Road, Parkville, Victoria, 3010, Australia; 4 National eResearch Collaboration Tools and Resources, Victoria, 3010, Australia; 5 ELIXIR-DE and de.NBI, Bioinformatics Group, Department of Computer Science, University of Freiburg, Freiburg, Germany; 6 ELIXIR-SE, National Bioinformatics Infrastructure Sweden (NBIS), Scilifelab, Department of Biochemistry and Biophysics (DBB), Stockholm University, Stockholm, Sweden; 7 ELIXIR-ES, Spanish National Bioinformatics Institute (INB), Spanish National Cancer Research Centre (CNIO), Calle de Melchor Fernández Almagro 3, Madrid, 28029, Spain; 8 Software Sustainability Institute, JCMB, University of Edinburgh, Edinburgh, EH9 3FD, UK; 9 Repositive Ltd, Future Business Centre, Cambridge, UK; 10 EMBL Australia Bioinformatics Resource, Lab-14, The University of Melbourne, 700 Swanston St, Parkville, Victoria, 3053, Australia; 11 EMBL-EBI, Wellcome Genome Campus, Hinxton, CB10 1SD, UK; 12 Barcelona Supercomputing Center, Barcelona, 08034, Spain; 13 Department of Biochemistry and Molecular Biomedicine, Universitat de Barcelona, Barcelona, 08028, Spain; 14 ELIXIR-UK, Software Sustainability Institute, School of Computer Science, University of Manchester, Oxford Road, Manchester, M13 9PL, UK; 15 Oxford e-Research Centre, University of Oxford, Oxford, UK; 16 BBMRI-ERIC, Neue Stiftingtalstraße 2/B/6, Graz, 8010, Austria; 17 Dutch TechCenter for Life Sciences and ELIXIR-NL, Utrecht, Netherlands; 18 ELIXIR-DK, Technical University of Denmark, Denmark, Denmark; 19 National Center for Supercomputing Applications, University of Illinois at Urbana-Champaign, Urbana, IL, USA; 20 School of Information Sciences, University of Illinois Urbana Champaign, Urbana, IL, USA; 21 Department of Electrical and Computer Engineering, University of Illinois Urbana Champaign, Urbana, IL, USA; 22 Department of Computer Science, University of Illinois Urbana Champaign, Urbana, IL, USA; 23 ELIXIR-SI, Faculty of Medicine, University of Ljubljana, Ljubljana, Slovenia; 24 DETI/IEETA, University of Aveiro, Aveiro, Portugal; 25 Center for Open Science, Charlottesville, VA, USA; 26 Stemformatics, University of Melbourne, Melbourne, Australia; 27 Computational Biology Division, Department of Integrative Biomedical Sciences, Institute for Infectious Disease and Molecular Medicine, University of Cape Town, Cape Town, South Africa; 28 ELIXIR-CZ, Faculty of Information Technology, Czech Technical University in Prague, Prague, Czech Republic; 29 BBMRI.at, Alpen-Adria-University Klagenfurt, Klagenfurt, Austria; 30 GRIB, Institut Hospital del Mar d’Investigacions Mèdiques (IMIM), Universitat Pompeu Fabra, Barcelona, Spain; 31 School of Agriculture, Food & Wine, University of Adelaide, Adelaide, Australia; 32 Central European Institute of Technology (CEITEC), Brno, Czech Republic; 33 National Centre for Biomolecular Research, Masaryk University, Brno, Czech Republic; 34 ELIXIR-EE, Institute of Computer Science, University of Tartu, Tartu, Estonia; 35 Science & Technologies Facilities Council, Swindon, UK; 36 Australian National Data Service, Melbourne, Australia; 37 Australian Genome Research Facility Ltd., Melbourne, Australia; 38 Centre for Language and Speech Technology, Radboud University Nijmegen, Nijmegen, Netherlands; 39 IBPM-CNR, Department of Biochemical Sciences , Sapienza University of Rome, Rome, Italy; 40 Faculty of Information Technology, Monash University, Victoria, Australia; 41 Software Sustainability Institute, Web and Internet Science, University of Southampton, Southampton, UK

**Keywords:** Open Source, code, software, guidelines, best practices, recommendations, Open Science, quality, sustainability, FAIR

## Abstract

Scientific research relies on computer software, yet software is not always developed following practices that ensure its quality and sustainability. This manuscript does not aim to propose new software development best practices, but rather to provide simple recommendations that encourage the adoption of existing best practices. Software development best practices promote better quality software, and better quality software improves the reproducibility and reusability of research. These recommendations are designed around Open Source values, and provide practical suggestions that contribute to making research software and its source code more discoverable, reusable and transparent. This manuscript is aimed at developers, but also at organisations, projects, journals and funders that can increase the quality and sustainability of research software by encouraging the adoption of these recommendations.

## Introduction

New discoveries in modern science are underpinned by automated data generation, processing and analysis: in other words, they rely on software. Software, particularly in the context of research, is not only a means to an end, but is also a collective intellectual product and a fundamental asset for building scientific knowledge. More than 90% of scientists acknowledge software is important for their own research and around 70% say their research would not be feasible without it ( Hannay *et al.*, 2009; Hettrick *et al.*, 2016).

Scientists are not just users of software; they are also prime producers ( Goble, 2014). 90% of scientists developing software are primarily self-taught and lack exposure and incentives to adopt software development practices that are widespread in the broader field of software engineering ( Wilson *et al.*, 2014). As a result, software produced for research does not always meet the standards that would ensure its quality and sustainability, affecting the reproducibility and reusability of research ( Crouch *et al.*, 2013).

Open Source Software (OSS) is software with source code that anyone can inspect, modify and enhance. OSS development is used by organisations and projects to improve accessibility, reproduction, transparency and innovation in scientific research ( Mulgan *et al.*, 2005; Nosek *et al.*, 2015). OSS not only increases discoverability and visibility, but it also engages developer and user communities, provides recognition for contributors, and builds trust among users ( McKiernan *et al.*, 2016). OSS development significantly contributes to the reproducibility of results generated by the software and facilitates software reusability and improvement ( Ince *et al.*, 2012; Perez-Riverol *et al.*, 2014). Opening code to the public is also an opportunity for developers to showcase their work, so it becomes an incentive for adoption of software development best practices ( Leprevost *et al.*, 2014). Thus, OSS can be used as a vehicle to promote the quality and sustainability of software, leading to the delivery of better research.

This manuscript describes a core set of OSS recommendations to improve the quality and sustainability of research software. It does not propose new software development best practices, but rather provides easy-to-implement recommendations that encourage adoption of existing best practices. These recommendations do not aim to describe in detail how to develop software, but rather lay out practical suggestions on top of Open Source values that go towards making research software and its source code more discoverable, reusable and transparent.

The OSS recommendations should be applied following existing and complementary guidelines like best practices, manifestos and principles that describe more specific procedures on how to develop and manage software. Some of these complementary guidelines are related to version control, code review, automated testing, code formatting, documentation, citation and usability. ( Artaza *et al.*, 2016; DagstuhlEAS, 2017; Gilb, 1988; Leprevost *et al.*, 2014; List *et al.*, 2017; Perez-Riverol *et al.*, 2016; Prlić & Procter, 2012; Smith *et al.*, 2016; Wilson *et al.*, 2014; Wilson *et al.*, 2016).

This manuscript also aims to encourage projects, journals, funders and organisations to both endorse the recommendations and to drive compliance through their software policies. The recommendations are accompanied by a list of arguments addressing common questions and fears raised by the research community when considering open sourcing software.

In this manuscript, software is broadly defined to include command line software, graphical user interfaces, desktop and mobile applications, web-based services, application program interfaces (APIs) and infrastructure scripts that help to run services.

## Target audience

Our target audience includes leaders and managers of organisations and projects, journal editorial bodies, and funding agencies concerned with the provision of products and services relying on the development of open research software. We want to provide these stakeholders with a simple approach to drive the development of better software. Though these OSS recommendations have mostly been developed within, and received feedback from, the life science community, the document and its recommendations apply to all research fields.

Strategies to increase software quality usually target software developers, focusing on training and adoption of best practices ( Wilson *et al.*, 2014). This approach can yield good results, but requires a significant effort as well as personal commitment from developers ( Wilson, 2014). For an organisation employing scientists and developers with different sets of programming skills and responsibilities, it is not easy to endorse specific best practices or define a broad range of training needs. It is easier to endorse a set of basic recommendations that are simple to monitor, simple to comply with, and which drive the adoption of best practices and reveal training needs. The OSS recommendations aim to create awareness, encourage developers to be more conscious of best practices, and make them more willing to collaborate and request support. The recommendations define broad guidelines, giving developers freedom to choose how to implement specific best practices.

In terms of the adoption of these recommendations, we see endorsement as the first step: that is, agreeing to support the OSS recommendations without a formal process for implementation. Promotion is a second step: that is, actively publicising and incentivising the OSS recommendations within the organisation as well as globally. Compliance is the third step: to formally implement them within the organisation, with ongoing monitoring and public reporting if possible. To facilitate progress, we propose that organisations, projects, journals, as well as funding agencies include these OSS recommendations as part of their policies relating to the development and publication of software.

Open Source Software is not just adopted by non-profit organisations, but also by commercial companies as a business model ( Popp, 2015). Therefore, we encourage not only publicly funded projects but also for-profit entities to adopt OSS and support these recommendations.

## Recommendations

### 1. Make source code publicly accessible from day one

Develop source code in a publicly accessible, version controlled repository (e.g., GitHub and Bitbucket) from the beginning of the project. The longer a project is run in a closed manner, the harder it is to open it later ( Fogel, 2005). Opening code and exposing the software development life cycle publicly from day one:

- Promotes trust in the software and broader project
- Facilitates the discovery of existing software development projects
- Provides a historical public record of contributions from the start of the project and helps to track recognition
- Encourages contributions from the community
- Increases opportunities for collaboration and reuse
- Exposes work for community evaluation, suggestions and validation
- Increases transparency through community scrutiny
- Encourages developers to think about and showcase good coding practices
- Facilitates reproducibility of scientific results generated by all prior versions of the software
- Encourages developers to provide documentation, including a detailed user manual and clear in-code comments

Some common doubts and questions about making software Open Source are discussed in the Supplementary File S1, “Fears of open sourcing and some ways to handle them”.

### 2. Make software easy to discover by providing software metadata via a popular community registry

Facilitate discoverability of the software project and its source code by registering metadata related to the software in a popular community registry. Metadata might include information like the source code location, contributors, licence, version, identifier, references and how to cite the software. Metadata registration:

- Increases the visibility of the project, the software, its use, its successes, its references, and its contributors
- Provides easy access for software packagers to deploy your software, thus increasing visibility
- Encourages software providers to think about the metadata that describes software as well as how to expose such metadata
- Helps to expose the software metadata in a machine readable format via the community registry
- Increases the chances of collaboration, reuse, and improvement

Examples of community registries of software metadata are bio.tools ( Ison *et al.*, 2016), ( Ison *et al.*, 2016) biojs.io ( Corpas *et al.*, 2014; Gómez *et al.*, 2013) and Omic Tools ( Henry *et al.*, 2014) in the life sciences and DataCite ( Brase, n.d.) as a generic metadata registry for software as well as data.

### 3. Adopt a licence and comply with the licence of third-party dependencies

Adopt a suitable Open Source licence to clarify how to use, modify and redistribute the source code under defined terms and conditions. Define the licence in a publicly accessible source code repository, and ensure the software complies with the licences of all third party dependencies. Providing a licence:

- Clarifies the responsibilities and rights placed on third parties wishing to use, copy, redistribute, modify and/or reuse your source code
- Enables using the code in jurisdictions where “code with no licence” means it cannot be used at all
- Protects the software’s intellectual property
- Provides a model for long-term sustainability by enabling legally well-founded contributions and reuse

We advise choosing a OSI-approved Open Source Licence unless your institution or project requires a different licence. Websites like “ Choose an open source license” provide guidelines to help users to select an OSI-approved Open Source Licence. Organisations like the OSS Watch also provide advice on how to keep track of the licences of software dependencies. For reusability reasons, we also advise authors to disclose any patents and pending patent applications known to them affecting the software.

### 4. Define clear and transparent contribution, governance and communication processes

Open sourcing your software does not mean the software has to be developed in a publicly collaborative manner. Although it is desirable, the OSS recommendations do not mandate a strategy for collaborating with the developer community. However, projects should be clear about how contributions can be made and incorporated by having transparent governance model and communication channels. Clarity on the project structure, as well as its communication channels and ways to contribute:

- Increases transparency on how the project and the software is being managed
- Helps to define responsibilities and how decision are made in the software project
- Helps the community know how to collaborate, communicate and contribute to the project

For instance the Galaxy project’s website describes the team’s structure, how to be part of the community, and their communication channels.

## Alignment with FAIR data principles

The FAIR Guiding Principles for scientific data management and stewardship provide recommendations on how to make research data findable, accessible, interoperable and reusable (FAIR) ( Wilkinson *et al.*, 2016). While the FAIR principles were originally designed for data, they are sufficiently general that their high level concepts can be applied to any digital object including software. Though not all the recommendations from the FAIR data principles directly apply to software, there is good alignment between the OSS recommendations and the FAIR data principles (see Table 1).

**Table 1.** Comparison between the OSS recommendations and the FAIR data principles ( Wilkinson *et al.*, 2016).

The FAIR Guiding Principles	OSS recommendations
To be Findable: F1. (meta)data are assigned a globally unique and persistent identifier; F2. data are described with rich metadata (defined by R1 below); F3. metadata clearly and explicitly include the identifier of the data it describes; F4. (meta)data are registered or indexed in a searchable resource	“R2. Make software easy to discover by providing software metadata via a popular community registry” aligns with the Findability principle, helping to increase visibility and helping software providers to think about how to describe software metadata (versions, identifiers, contributors, citations, etc.)
To be Accessible: A1. (meta)data are retrievable by their identifier using a standardized communications protocol; A1.1 the protocol is open, free, and universally implementable; A1.2 the protocol allows for an authentication and authorization procedure, where necessary; A2. metadata are accessible, even when the data are no longer available	“R1. Make source code publicly accessible from day one” focuses on openness including accessibility. The FAIR accessible principle instead opens the door to data that is restricted access e.g. for privacy reasons. Since such reasons do not apply for software, the OSS recommendations prefer to direct towards openness instead, supporting open science to the maximum extent.
To be Interoperable: I1. (meta)data use a formal, accessible, shared, and broadly applicable language for knowledge representation; I2. (meta)data use vocabularies that follow FAIR principles; I3. (meta)data include qualified references to other (meta)data	This OSS recommendations do not aim to address software interoperability directly but contribute to a more homogenous description of software by encouraging software providers to register software metadata into registries providing specific metadata guidelines.
To be Reusable: R1. meta(data) are richly described with a plurality of accurate and relevant attributes; R1.1. (meta)data are released with a clear and accessible data usage license; R1.2. (meta)data are associated with detailed provenance; R1.3. (meta)data meet domain-relevant community standards	“R3. Adopt a license and comply with the licence of third-party dependencies” aligns with the Reusability principle, helping to define to what extent the source code can be used and reused by the community, as a standalone software or as part of other software. Open availability of tools and libraries working with data formats can be a great help in making data interoperable: e.g. reuse of the same tools to read and write data can prevent subtle interoperability problems. Reproducibility of experiments and reuse of data is facilitated by the open availability of the associated software which is part of the provenance. All of the OSS recommendations thereby facilitate data Reusability.

There are also distinctions between the OSS recommendations and the FAIR data principles. The FAIR data principles have a specific emphasis on enhancing machine-readability: the ability of machines to automatically find and use data. This emphasis is not present in the OSS recommendations which expect machine readable software metadata to be available via software registries. The OSS recommendations are less granular and aim to enhance understanding and uptake of best practices; they were designed with measurability in mind. The FAIR data principles do not have such built-in quantification yet. FAIR metrics are a separate effort under development, lead by the Dutch Techcentre for Life Sciences ( Eijssen *et al.*, 2016).

The community registries can play an important role in making software metadata FAIR by capturing, assigning and exposing software metadata following a standard knowledge representation and controlled vocabularies that are relevant for domain-specific communities. Thus we expect the community registries to provide guidelines on how to provide software metadata following the FAIR Guiding Principles ( Wilkinson *et al.*, 2016).

## Conclusion

The OSS recommendations aim to encourage the adoption of best practices and thus help to develop better software for better research. These recommendations are designed as practical ways to make research software and its source code more discoverable, reusable and transparent, with the desired objective to improve its quality and sustainability. Unlike many software development best practices tailored for software developers, the OSS recommendations aim to target a wider audience, particularly research funders, research institutions, journals, group leaders, and managers of projects producing research software. The adoption of these recommendations offer a simple mechanism for these stakeholders to promote the development of better software and an opportunity for developers to improve and showcase their software development skills.

## Supplementary material

Supplementary File 1: ‘Fears of open sourcing and some ways to handle them’. In this appendix we aim to expose some of the common fear scenarios related to open sourcing, and some ways to handle them.
