# Supplementary material for: Four simple recommendations to encourage best practices in research software
Source: F1000Res. 2017 Jun 13;6:ELIXIR-876. [Version 1] doi: 10.12688/f1000research.11407.1 (PMC5490478; doi:10.12688/f1000research.11407.1)
Supplement: Supplementary file 1 [file f1000research-6-12314-s0000.tgz › 21d372e3-6e9f-4dfd-99df-415c8c51fdec.docx]

# Fears of open sourcing and some ways to handle them

Although the scientific community is used to collaboration, competition is an established practice and drives many decisions on how to share information. It has been a trend to keep data and other research artefacts like software private, even after research results get published. Thus, it is not surprising that many developers involved in research do not proactively release software as Open Source [(Stodden, 2009)](https://paperpile.com/c/Gzhyvy/EYsB). Though part of the fear might be justified, Open Source Software comes with more advantages than disadvantages, and some fears arise from a misunderstanding of the risks involved. This supplementary file aims to expose some of the common fear scenarios related to open sourcing and proposes some ways to handle them. The scenarios have been grouped into two sections. The first one collects hypothetical fears of principal investigators and other managerial positions involved in the decision making of how software is developed. The second section includes hypothetical fears of software developers.

## Principal Investigator Perspective

**My group’s software and its unique methodology gives our research a competitive edge that will be lost if we released our code in the open.**

- Open sourcing your software gives others the opportunity to identify issues and improvements, and addressing these may make you more competitive.
- People with expertise will be more interested in understanding the software and contributing to developing existing functionality further, than in replicating it. However, if the software and its methodology is not open it provides an excuse and encourages people to duplicate it.
- If a methodology or functionality has been developed, and it is available in an existing source code repository, it will discourage developers to replicate it. Instead, it will encourage them to reuse it and through proper acknowledgement this should benefit the original developers.
- Research is far more important than the software that facilitates it, and the software usually lags behind the underlying research ideas.

**It’ll be impossible to make software developed in collaboration with commercial companies and industry Open Source because it will be too complicated to resolve the legal issues**

- You could consult an Intellectual Property (IP) specialist such as [OSS-Watch](http://oss-watch.ac.uk/).
- Using an [Open Source Initiative approved license](https://opensource.org/licenses) will make it easier as commercial IP legal departments recognise them.

**Preparing my code to be Open Source will take a lot of time and effort**

- The expected time benefits often outweigh the initial investment. e.g. [Taverna transition to the Apache Incubator](https://taverna.incubator.apache.org/).
- It will help your internal development team in their adoption of best practices and in the long run will save time and effort.
- Publishing code in a publicly accessible, version controlled repository does not require much effort and can be done in few minutes. Developers should not be afraid to publish existing code as it is and improve it later if necessary.
- Funders are more supportive with open practices and might encourage to support initiatives that develop open source software.
- You might receive contributions from the community, such as issues reporting after testing, patches for the code, etc. which will contribute to your effort and save you time.

**An open source license will put off companies**

- Many companies use Open Source software - they care about functionality and support. Examples include: [Python](https://www.python.org/), [Perl](https://www.perl.org/), [RDKit](http://www.rdkit.org/), [Linux](https://www.linux.org/), [Docker](https://www.docker.com/) and [Apache HTTP server](https://httpd.apache.org/).

**I will have to support users who might or not be in my field of work**

- Open sourcing your software does not mean the software has to be developed in a publicly collaborative manner, although doing so can help foster collaboration and reputation in your own or related fields, which can lead to new scientific collaborations and funding.
- Having an impact outside of your own field can prove valuable to research funders.
- You are probably being affected by the same issues that those users want fixed.
- You can resolve issues early that may affect your own research in the future.

**I will be liable or my reputation will suffer if someone uses my software incorrectly, e.g. if someone publishes a paper based on results that use my software, but then if the paper must be withdrawn or retracted, my software may be blamed**

- A good OSI-approved license will disclaim/waive liability better than having no license or an unexamined academic license [(Stodden 2009)](https://paperpile.com/c/Gzhyvy/oO5w).
- Whether it’s open or not, software is often distributed and used by other people.
- Improve your documentation to help avoid misuse. This will also help manuscript reviewers and the rest of the research community identify inappropriate use.

**I will have to pay for the cost of infrastructure**

- There are many providers who allow you to use their resources for free if you are an open source project. Eg. [GitHub](https://github.com/) offers for free version control repositories for hosting open-source projects.
- Continuous Integration testing, hosting of documentation, test coverage calculation, DOI are all given for free to Open Source projects. Hosting your own infrastructure is expensive, also in human resources.
- Opening your source code does not require infrastructure on your part, you can just upload it to a repository hosting site like [GitHub](https://github.com/) and [BitBucket](https://bitbucket.org/).

**People will scoop us**

- Unnecessary duplication of efforts and scooping ideas is negatively perceived by the research community. Open source makes your ideas and code more visible and accessible to the community. Thus, open source might discourage more than encourage scooping.
- If your work and ideas are online and have a timestamp you can always prove that they were actually yours.
- Just because your software is used by others, doesn't mean you will be scooped. For instance in biological sciences Software Projects like [BioPython](http://biopython.org/) and [OpenBabel](http://openbabel.org/wiki/Main_Page) are big players in industry, and they published their work years after the first public releases.

## Developer Perspective

**My software is such a bunch of hacks that no-one will want to contribute to it**

- If it is closed, no-one can contribute to it anyway. If it is open, then people could still contribute, even if the code quality is not great.
- Even inspiration *(how things were done once)* is worth sharing.
- If your software is useful, others will convert these bunch of hacks into proper code.

For more information, please see the software sustainability institute’s ‘[Top ten reasons to not share your code (and why you should anyway)](https://www.software.ac.uk/blog/2016-10-07-top-ten-reasons-not-share-your-code-and-why-you-should-anyway)’.

**I am the expert in this field. I am the best person to build this software and do not see how letting other people contribute will help my software**

- You may be the expert in your field, but the software is just a tool you are building. Different people with different skillsets can contribute to different aspects of your software like usability, testing, documentation, etc.
- Providing open code and open training might help others to become experts as well.
- Documentation should help train new contributors: code comments, usage, development setup.

**Someone takes my software and sets it up as a service, but wrongly. This would reflect badly on my original software (e.g. they used the wrong data).**

- You can use trademark enforcement of the name to make it clear that this is not your or your software’s responsibility.
- You can avoid this situation following best practices. For instance, by documenting your setup, keeping communication channels in order or automating your software packaging with software containers or similar tools.

**To be able to use my software, other people need the ‘right data’, and I cannot give this to them because it is protected or confidential, so my software is of no use**

- You can provide simulated or reference data which you should have in any case to help with testing.
- Your software may be adopted and modified to make it suitable for the types of data you might not have considered.

**I should be doing research, not spending time open sourcing software**

- Science is fundamentally based on accuracy and trust in results. Open Source code can be more trusted than black box solutions (Morin et al. 2012).
- If you are very busy, just open up what you already have or are working on. You can easily create a GitHub account and upload your code following few steps.
- You can carry out research more efficiently if you reuse software from others, so it is only fair to give back to the community to enable somebody else to save time too.

**I will not receive any credit for my work**

- Open sourcing has been shown to improve citation and research impact in communities
- Consider publishing your work in a journal that accepts publication of software tools. For example ‘The Journal of Open Source Software’ or ‘The Journal of Open Research Software’.

**I will not be able to publish a paper about my research or about my software**

- Academic credit in scientific research relies primarily on peer-reviewed publications. The software sustainability institute lists [several journals that accept submissions which are primarily about the software](https://www.software.ac.uk/which-journals-should-i-publish-my-software), and not necessarily about the new algorithms or new research. The community also recognises the value of publishing preprints like [arXiv.org](https://arxiv.org/).

# References

[Morin, A., J. Urban, P. D. Adams, I. Foster, A. Sali, D. Baker, and P. Sliz. 2012. “Shining Light into Black Boxes.” *Science* 336 (6078): 159–60.](http://paperpile.com/b/Gzhyvy/9OgP)

[Stodden, Victoria. 2009. “Enabling Reproducible Research: Open Licensing for Scientific Innovation,” March.](http://paperpile.com/b/Gzhyvy/oO5w) <https://papers.ssrn.com/abstract=1362040>[.](http://paperpile.com/b/Gzhyvy/oO5w)
