# Supplementary material for: Four simple recommendations to encourage best practices in research software
Source: F1000Res. 2017 Jun 13;6:ELIXIR-876. [Version 1] doi: 10.12688/f1000research.11407.1 (PMC5490478; doi:10.12688/f1000research.11407.1)
Supplement: Peer review history

# Peer review: Referee response for version 1

**DOI:** 10.5256/f1000research.12314.r23464

**Published:** 2017-07-10

**Authors:** Stefanie Betz

**Affiliations:** 1 Karlsruhe Institute of Technology (KIT), Karlsruhe, Germany

The article advocates research software openness presenting four recommendations to improve research software visibility, re-usability, and transparency. I really like the article and I think it is important to open research software. Please find below my feedback. (Overall, I agree with the comments of Milad Miladi):

- In my opinion, the title does not reflect the content of the article. The focus is on adopting OSS and supporting the provided four recommendations to help to develop better software for better research. Currently, only the second part (supporting the provided four recommendations to help to develop better software for better research) is reflected in the title not the part about OSS.
- I am not sure everybody is familiar with the open registry platforms. Thus, some more information regarding them or a link to background information would be nice.
- I think recommendation four should include documentation.

I have read this submission. I believe that I have an appropriate level of expertise to confirm that it is of an acceptable scientific standard.

# Peer review: Referee response for version 1

**DOI:** 10.5256/f1000research.12314.r23467

**Published:** 2017-06-26

**Authors:** Greg (Gregory V.) Wilson

**Affiliations:** 1 Rangle.io, Toronto, ON, Canada

The article presents focused, well-argued advocacy for improving software development practices in the sciences. None of the recommendations will be surprising to those already involved in open science, but as only a small minority of researchers actually *do* them, it is worth presenting them forcefully and succinctly. I would recommend shortening the introductory material (sections "Introduction" and "Target Audience"), but that is a minor point.

I have read this submission. I believe that I have an appropriate level of expertise to confirm that it is of an acceptable scientific standard.

# Peer review: Referee response for version 1

**DOI:** 10.5256/f1000research.12314.r23468

**Published:** 2017-06-26

**Authors:** Roberto Di Cosmo

**Affiliations:** 1 Software Heritage, Inria, Rocquencourt, France; 2 Paris Diderot University, Paris, France

This article presents four simple recommendations that may improve the overall quality and visibility of research software. This reviewer agrees with the basic principles set forth by the authors, and hopes they will be widely shared and adopted at least for software that is expected to last longer than the time it takes for the corresponding research paper to be accepted and/or presented.

I have read this submission. I believe that I have an appropriate level of expertise to confirm that it is of an acceptable scientific standard.
